# EnRICH: **E**xtractio**n **and **R**anking using **I**ntegration and **C**riteria **H**euristics

**DOI:** 10.1186/1752-0509-7-4

**Published:** 2013-01-15

**Authors:** Xia Zhang, M Heather West Greenlee, Jeanne M Serb

**Affiliations:** 1 Department of Ecology, Evolution, and Organismal Biology, Iowa State University, Ames, Iowa, USA; 2 Department of Biomedical Sciences, 2008 Veterinary Medicine, Iowa State University, Ames, IA 50010, USA; 3 Interdepartmental Genetics Program, Iowa State University, Ames, Iowa, USA; 4 Bioinformatics and Computational Biology Program, Iowa State University, Ames, Iowa, USA

**Keywords:** Qualitative integration, High-throughput data, Heterogeneous data, Network, Network visualization, Candidate prioritization

## Abstract

**Background:**

High throughput screening technologies enable biologists to generate candidate genes at a rate that, due to time and cost constraints, cannot be studied by experimental approaches in the laboratory. Thus, it has become increasingly important to prioritize candidate genes for experiments. To accomplish this, researchers need to apply selection requirements based on their knowledge, which necessitates qualitative integration of heterogeneous data sources and filtration using multiple criteria. A similar approach can also be applied to putative candidate gene relationships. While automation can assist in this routine and imperative procedure, flexibility of data sources and criteria must not be sacrificed. A tool that can optimize the trade-off between automation and flexibility to simultaneously filter and qualitatively integrate data is needed to prioritize candidate genes and generate composite networks from heterogeneous data sources.

**Results:**

We developed the java application, EnRICH (**E**xtractio**n** and **R**anking using **I**ntegration and **C**riteria **H**euristics), in order to alleviate this need. Here we present a case study in which we used EnRICH to integrate and filter multiple candidate gene lists in order to identify potential retinal disease genes. As a result of this procedure, a candidate pool of several hundred genes was narrowed down to five candidate genes, of which four are confirmed retinal disease genes and one is associated with a retinal disease state.

**Conclusions:**

We developed a platform-independent tool that is able to qualitatively integrate multiple heterogeneous datasets and use different selection criteria to filter each of them, provided the datasets are tables that have distinct identifiers (required) and attributes (optional). With the flexibility to specify data sources and filtering criteria, EnRICH automatically prioritizes candidate genes or gene relationships for biologists based on their specific requirements. Here, we also demonstrate that this tool can be effectively and easily used to apply highly specific user-defined criteria and can efficiently identify high quality candidate genes from relatively sparse datasets.

## Background

Hundreds to thousands of candidate genes, or genes of interest, can now be generated from a single experiment utilizing high throughput screening technologies. However, the number of candidate genes that can be experimentally studied in-depth is often constrained by time and cost. Therefore, prioritization of candidate genes is a critical step in the experimental process. Approaches to identify ‘the most promising’ candidates are becoming increasingly more sophisticated. For example, when microarray studies were initially reported, ‘the most promising’ candidates were often the most differentially expressed and could be obtained by a simple ranking of candidates based on fold change. As more data has become available, biologists have begun to look for ways [1-4] to use multiple data sources to increase the accuracy of candidate gene prioritization. Some tools have already been developed to address this need [5-11]. These tools prioritize candidates by their similarity to genes already known to be important for a particular biological process (e.g., genes known to regulate cell cycle in yeast). Multiple data sources including published literature, gene sequence, functional annotation, etc. can be considered when comparing the similarity of candidates to ‘known genes’. These tools [5-11] have made important progress towards the problem of candidate prioritization. However, these tools use data queried from predetermined sources, such as public databases, and include embedded criteria. Thus, these software packages have limited utility.

Biologists, with expertise in a given area, generally already have a list of criteria that could be applied to identify high quality candidates. Likely, for a given set of experiments and resulting datasets, the best candidates may satisfy one set of criteria in one dataset and a separate set of criteria in another dataset. Currently, there is no tool that allows simultaneous consideration of heterogeneous datasets to identify candidates that satisfy multiple criteria. This problem does not only relate to candidate genes, but also to putative relationships between genes in networks.

Putative gene relationships can be inferred from many heterogeneous sources (e.g., physical interactions, genetic interactions, expression correlation and interactions predicted by computational models). While each of these relationships from a given dataset should be interpreted differently (and subject to very different criteria), the ability to easily hypothesize gene relationships based on their meeting appropriate criteria in multiple datasets is an attractive prospect. This task not only calls for an automated filtering and integration tool, but also demands great flexibility of data sources and the ability to set filtering criteria. Finally, for proper interpretation, visualization of the resulting network must facilitate inspection by 1) retaining the original data sources of each putative relationship and 2) providing a mechanism to easily manage the size of the displayed network. While some tools have been developed to generate composite networks from multiple data sources (e.g., the Cytoscape [12] plugin CABIN [13], GraphWeb [14] and GeneMania [15]), they do not fully address the problems stated above. For example, CABIN supports only one filter for a single source network and thus multiple criteria cannot be applied. GraphWeb [14] does not support filtering by user-defined criteria and interactive network visualization. GeneMania [15] helps to predict the function of a set of input genes by utilizing functional association data to generate a functional relevant network, but does not address integration of user-determined data and filtration with user-defined criteria.

We identified the need for a tool that is able to: 1) filter individual datasets using appropriate criteria and then integrate them to prioritize candidates that meet the criteria in multiple datasets; 2) allow users to define the most appropriate datasets and filtering criteria; and 3) provide an interactive visualization to facilitate the generation of an integrated network with a manageable size and connectedness. To address the open demand of filtering and qualitative integration of heterogeneous datasets, we have developed a stand-alone, portable and flexible java application with its own user-interactive visualization. EnRICH (**E**xtractio**n** and **R**anking using **I**ntegration and **C**riteria **H**euristics) will assist biologists in prioritization of genes and gene relationships from heterogeneous-source data.

## Implementation

EnRICH was implemented in Java (SE 6 JDK). EnRICH visualization was written in Processing (http://processing.org/), an open-source programming language to create images, animation and interactions. The separation of non-visual and visual modules of EnRICH lays a flexible foundation for future development and provides the user easy access to both the text and visual output results.

### Design

The objectives of EnRICH are firstly to provide a tool for integration of multiple or heterogeneous data sets to prioritize candidate molecules that fulfill user-defined criteria, and secondly to make the integration process flexible and simple for biologists who have little programming skill. Our aim-oriented design principles are 1) user-defined data sources and criteria, 2) simplicity which allows straight-forward application of user-defined criteria to filter user-defined datasets, and 3) platform independence.

**Figure 1:**
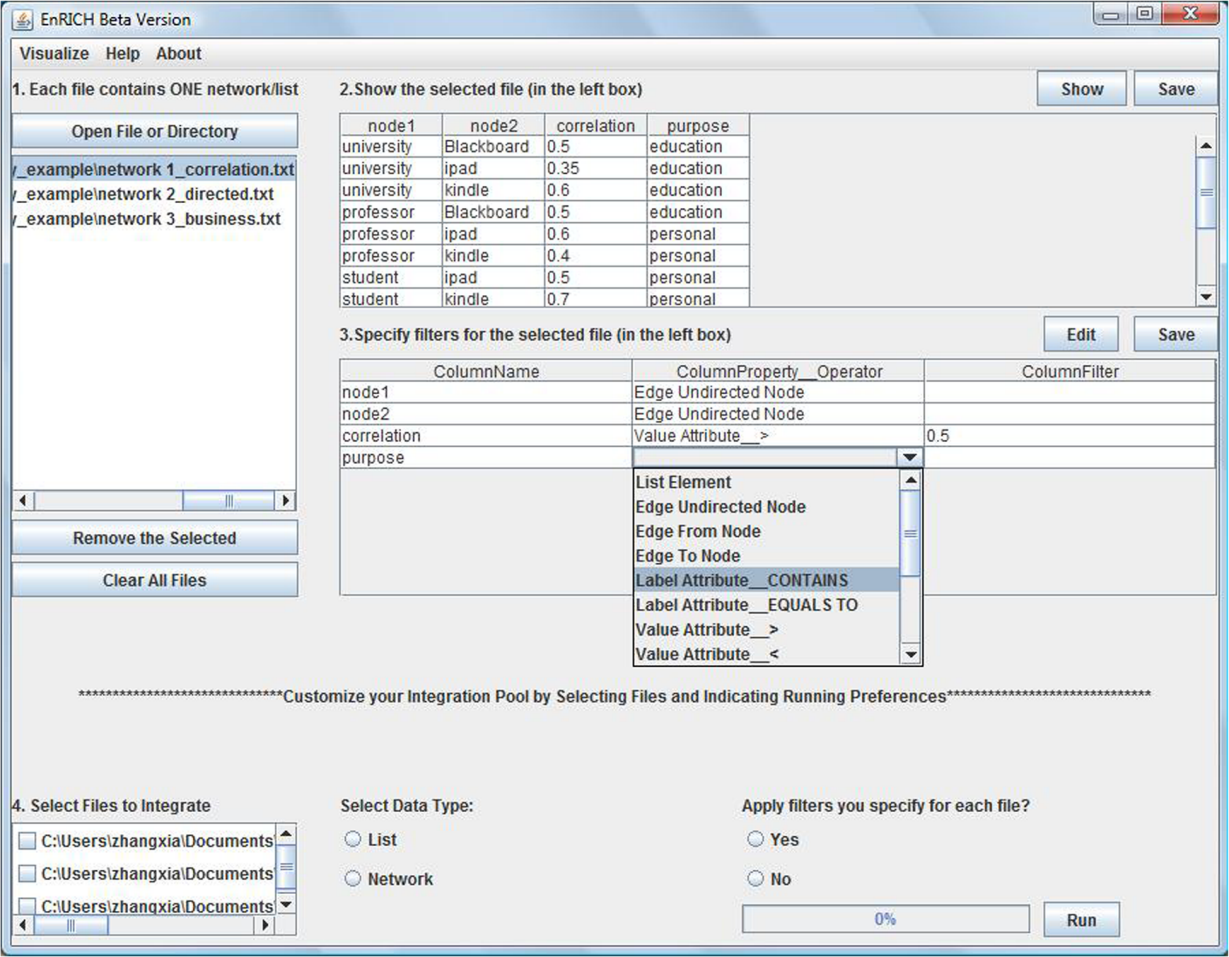
**EnRICH graphical user interface. **There are four major components in the user interface, which are numbered as 1, 2, 3 and 4. Component 1: upload input data; Component 2: browse or edit the selected file; Component 3: specify filtering criteria of the selected file; Component 4: select files to define integration pool.

The overall architecture of EnRICH is reflected in its workflow-like graphical user interface (GUI) (Figure 1). The first component (numbered as step 1 in the GUI) accepts a single file or a directory of files as input data and lists all files that can be selected for analysis. The second component (numbered as step 2 in the GUI) allows the user to display the selected file as a table and edit the table. The third component (numbered as step 3 in the GUI) enables the user to specify filtering criteria for each attribute of the selected file. The fourth component (numbered as step 4 in the GUI) displays all uploaded files for the user to customize an integration pool. It also provides the user running options on whether to apply filters that are already specified in step 3. The fifth component is a dialog window, which appears when the integration run is finished, and gives the user the option to save or visualize the result. For network data, EnRICH has an additional visualization component where the user can do an interactive visual analysis of the integrated network.

### Input data

The current version of EnRICH accepts two types of data: list and network. A list is a set of elements that could be genes, proteins, etc., which have their own unique identification code or name. List data can come from a large variety of sources. For example, a list of genes can be differentially expressed genes (DEGs) from the analysis of a microarray experiment, genes identified by genome-wide association mapping, or genes retrieved from a database query. Each list member may have one or more attributes. For example, each gene in a list of DEGs has its own significance value, functional annotation, etc. For EnRICH, list data is represented as a named matrix that is composed of one column of elements and zero to multiple attribute columns. Attributes can either be value attributes that will be taken as mathematical values or label attributes treated as tags.

A network is a set of nodes that are interconnected by edges representing particular relationships between nodes. Like list data, network data can originate from heterogeneous sources including yeast two-hybrid experiments, computational or statistical inferences, literature summaries or database queries. Although there are several standard languages or formats for network representation, we assume that biologists may not be familiar with those standards. Thus, EnRICH applies a popular node-pair/edge list format as the input format for network data, where an edge is denoted by the pair of nodes it connects. In the matrix format, network edges are represented by two columns of node names. Like list data, network edges may have values and label attributes. Accordingly, a network is a named matrix consisting of two columns of nodes and zero to multiple attribute columns. EnRICH allows blank fields in the attribute column when data are missing.

### Running mode

EnRICH runs in two modes: undefined (without filters) and defined (using specific criteria to filter attributes). The undefined mode simply ignores the attributes of networks or lists. Each list or network is considered as a source, and all sources will be merged together. The defined mode simultaneously considers integration of networks or lists as well as user-defined criteria (which filters out elements that do not meet the criteria) over each network or list. For both types of running modes, candidates (edges of a network or elements of a list) are ranked by their reoccurrence across all sources after integration. The filtering process is completely user-defined. Because the filter is totally attribute-based, the user sets filters most appropriate for their biological question, which may include a combination of filters for each attribute, and even multiple filters for multiple attributes. For example, two of the comparison operators (<, <=, >, >=, ==) applied at the same time can be used to set a cutoff range for value attributes or several tags can be used (with an OR operator between them) when the user wants to select multiple label values (e.g. two annotations) for one attribute. When there are multiple attributes, multiple filters (with an AND operator between them) can be applied simultaneously.

### Text output

EnRICH saves output results as a tab-delimited text file. In the output text file, the user can see what files were integrated, which filters were applied to each file, and the result. For list data, the result is a table, which consists of three columns: the label of an element, its reoccurrence across all lists, and names of source-lists. For network data, the result includes four tables: node statistics, edge statistics, nodes, and edges. The node degree reveals topological importance of the node, so the table of node statistics contains two columns, one column is the node degree (the number of connections a single node has) and the other is the number of nodes that are greater than or equal to (>=) this node degree. For the table of edge statistics, one column is edge reoccurrence (the number of times a single edge is recovered across all datasets) and the other is the number of edges that have an edge reoccurrence that are greater than or equal to (>=) this edge reoccurrence. The table of nodes and the table of edges are quite similar. Each has a column of nodes/edges, their reoccurrence, and source-networks. The only difference between the node and edge tables is that a node is represented by the node label and an edge is denoted as two node labels. The table of edges is a tab-delimited data table composed of several columns such as node label name, edge reoccurrence and source. Therefore, if desired, the user can directly copy or import them into another network visualization tool such as Cytoscape [12,16].

### Visualization

**Figure 2:**
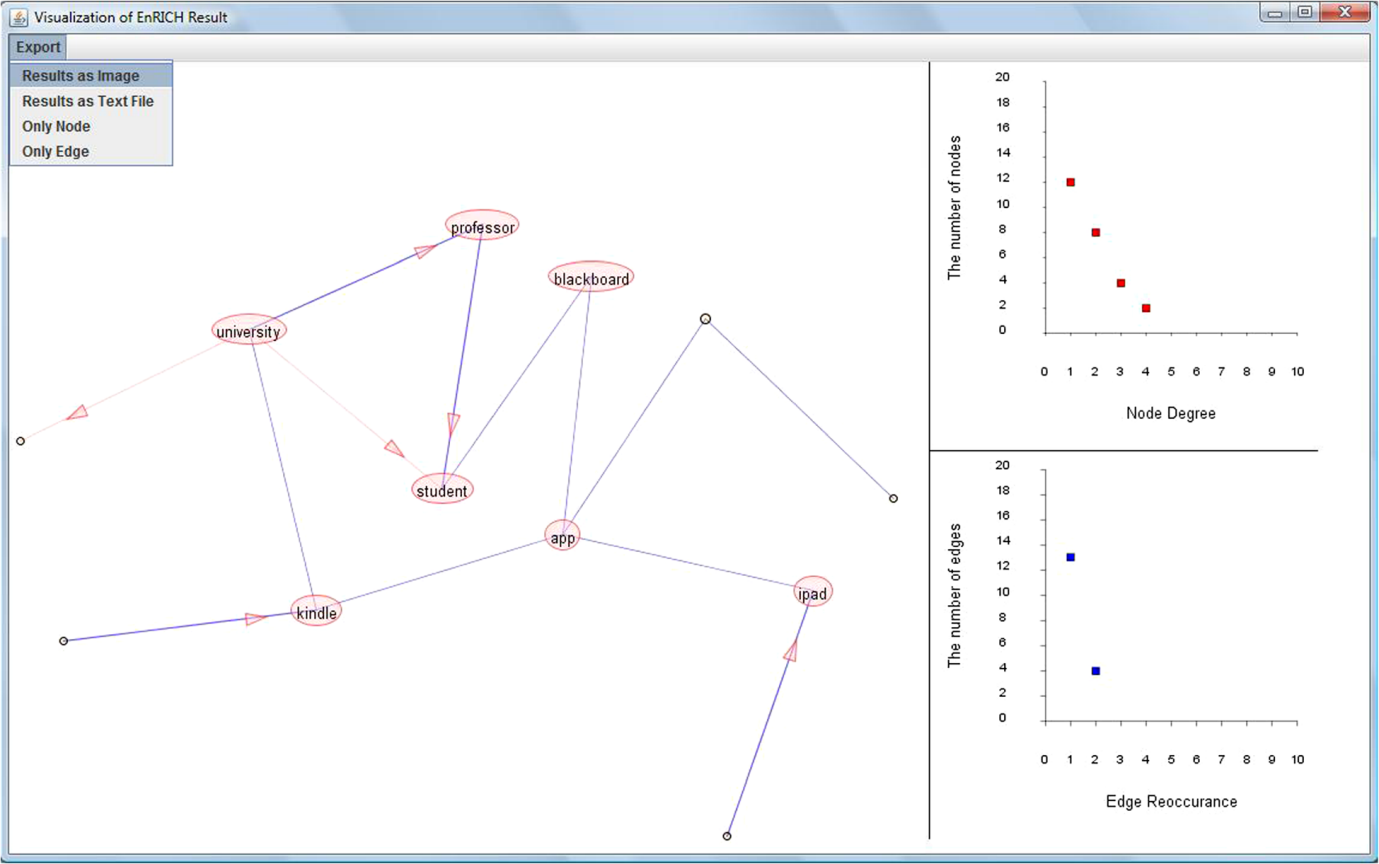
**EnRICH visualization window. **Left: integrated network from synthesizing data. Circles represent nodes, while circle size represents node degree (the number of connections one node has). Lines represent edges of a network and line stroke represents the amount of edge reoccurrence (the number of times one edge is recovered across data sources). Undirected edges are represented by blue lines, while directed edges are pink lines with pink arrows. The merged edge of undirected and directed data is denoted by blue line with a pink arrow. Right: top panel is the statistical plot of node degree vs. the number of nodes. Bottom panel is the statistical plot of edge reoccurrence vs. the number of edges. In the software, all data points in the two plots are clickable to update visualization of the integrated network.

EnRICH enables an interactive visual analysis of the integrated network without depending on a third-party visualization software. EnRICH network visualization consists of two components for user interaction: the integrated network and the plot of network statistics (Figure 2). In the integrated network, an undirected edge is drawn as a blue line while a directed edge is drawn as a pink line with a pink arrow to indicate the direction of the interaction (e.g. transcriptional regulation). A blue line with a pink arrow is used to denote merged undirected and directed edges. All edges and nodes can be repositioned, without changing connections, by clicking and then dragging the item on the screen. In addition, the user can click to show or dissipate node labels and edge sources at the node- and edge-specific level, instead of the whole network level. The plot component has two plots: 1) the number of nodes vs. node degree plot and 2) the number of edges vs. edge-reoccurrence plot. The number of nodes and the number of edges are two aspects of the network size, while the node degree reveals topological importance of node. Edge reoccurrence is the number of times the edge is recovered in different data sources, which implies the reliability of an edge. In conjunction, the two plots are used to balance the visualization of network size and quality. All data points in the two plots are clickable to re-draw the integrated network at the selected level of node degree or edge reoccurrence. This interactive plot gives the user an easily visible comparison of node degree, edge reoccurrence and network size, and allows the user to simultaneously visualize the network at corresponding levels. EnRICH also allows the exportation of the network as an image file in TIFF format, which is widely supported.

## Result and discussion

### Application of EnRICH

Retinal disease genes are genes that, when knocked out or mutated, cause retinal degeneration (https://sph.uth.tmc.edu/retnet/disease.htm). The identification of retinal disease genes is a major goal of retinal degenerative disease research and as part of this effort, there have been a significant number of experiments that describe transcriptional changes during normal retinal development [17-23]. Here, we present a case study in which we use EnRICH to integrate multiple gene lists to identify potential retinal disease genes.

*Nrl*[24-27] is a retinal disease gene that is associated with the retinal degenerative disease enhanced s-cone syndrome [28]. When *Nrl* is mutated, the resulting phenotype is an abundance of s-cone photoreceptors at the expense of rod photoreceptor differentiation [25,29], leading to the eventual death of all photoreceptors. During normal development, *Nrl* influences the cone versus the rod cell fate decision by activating rod-specific genes, including the genes *Rho* and *Nr2e3*[30]. *Rho*[31-33] is a rod-specific gene, the mutation of which leads to rod photoreceptor cell death and retinal degeneration. *Nr2e3*[34,35] is also essential during retinal development, as it promotes the expression of rod-specific genes (including *Rho*) and represses the expression of cone-specific genes in rods. The mutation of *Nr2e3* also causes enhanced s-cone syndrome [36]. Based on the known regulatory relationships between these three disease genes and their importance for normal photoreceptor development, we rationalize that the behavior of these genes would make good criteria to identify additional retinal disease genes.

Using these assumptions, we defined the following criteria to identify retinal disease genes: 1) candidates must be highly co-expressed with *Nrl*, *Nr2e3* and *Rho* during rod photoreceptor development of wild-type mice; and 2) candidates must be disregulated when *Nrl* is knocked out (as *Nr2e3* and *Rho* are). With these criteria in mind, we decided to use a microarray dataset [17] (GSE4051), which profiles gene expression in isolated rod photoreceptors at multiple developmental stages (E16, P2, P6, xP10, 4-weeks) in both *Nrl*-knockout and wild-type mice. In these microarrays, we confirmed that *Nr2e3* and *Rho* are highly co-expressed with *Nrl* in wildtype and are no longer co-expressed in the *Nrl* mutant.

**Figure 3:**
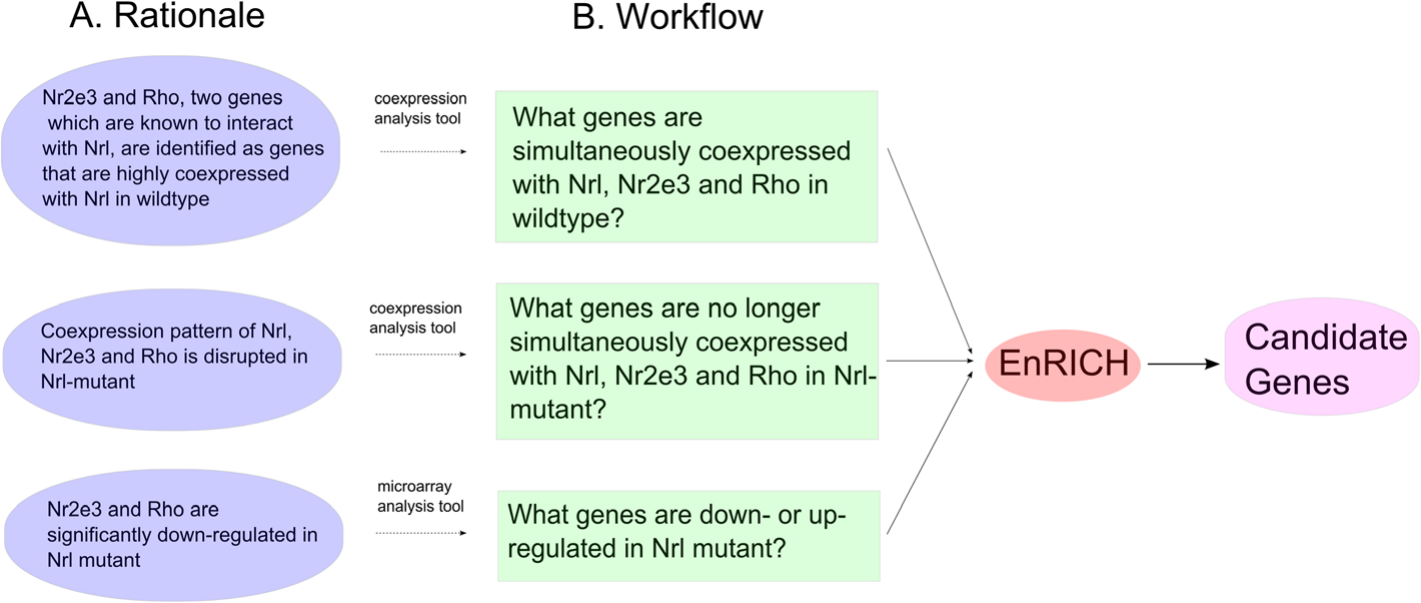
**Case study workflow. A. **Rationale illustrates our current knowledge of Nrl, Nr2e3 and Rho and their behaviors in the dataset we analyzed. **B. **Workflow displays the steps that we must go through in order to apply our criteria to identify candidate genes. These steps are executed by EnRICH to obtain candidate genes. Workflow is the implementation of the investigation process based on the rationale (A).

According to the corresponding workflow (Figure 3), we prepared, and subsequently integrated, three types of gene lists which are: Type 1) Genes that are co-expressed with *Nrl*, *Nr2e3* and *Rho* in developing wild type rod photoreceptors; Type 2) Genes that are co-expressed with *Nrl*, *Nr2e3* and *Rho* in developing photoreceptors isolated from *Nrl*-mutant retinas; and Type 3) Differentially expressed genes (DEGs) at each age when comparing gene expression in wild-type rod photoreceptors to *Nrl*-knockout rods. Each list contained attributes that were used to apply criteria filters (i.e. pairwise correlations for type 1 and 2, age at which expression was up or down regulated for type 3). To carry out the workflow, we first specified filtering criteria for each list. This is a key element of EnRICH, where users can simultaneously query multiple datasets to generate an ‘integrated result’. For this experiment, eight list datasets were integrated. The filtering criterion for six of the lists was an absolute value of the correlation coefficient greater than 0.9, while the filtering criterion for the two differentially expressed gene lists was the developmental time points P6 and P10 (for criteria on each single list, see Additional file 1: Table S1). Candidates that satisfied these filtering criteria in eight lists were identified as the highest priority candidates. All the lists were prepared from by standard analyses of the dataset GSE 4051 (calculation of co-expression coefficients within a genotype and differentially expressed genes between genotypes).

The execution of our workflow generated five candidate genes (see Additional file 2: Table S2) from an initial pool of 272 unique differentially expressed genes (see Additional file 3). Based on a literature/database search, four of our five candidate genes (*pde6b*, *gnb1*, *guca1a* and *cgna1*) are confirmed retinal disease genes [37-46], and the fifth gene (*kcne2*) has been shown to be up regulated during a neuroinflamatory response in the retinas of diabetic rats [47], making it a reasonable candidate for a disease gene as well. Thus, in our example analysis to identify disease genes, 80% of our candidates are known disease genes, while the remaining candidate has a demonstrated tie to the diseased retina, and is therefore a high quality candidate. Using a Fisher test we also concluded that retinal disease genes are significantly overrepresented in the genes prioritized by EnRICH, compared with genes not prioritized by EnRICH (see Additional file 3).

Our case study demonstrates that a well-conceived data integration and criteria-based filtration, as implemented in EnRICH, can effectively identify a limited number of high quality candidate genes for careful hypothesis-based investigation. Conversely, if the number of candidates returned is too small, slight adjustments in the filtration criteria may be easily made to generate a larger, while still reasonably-sized, candidate pool.

## Conclusions

EnRICH is a free java application which can qualitatively integrate results from large, heterogeneous data sources while simultaneously applying filters to each of them. It allows the user to define data sources, and to integrate them as well as specify multiple sorting criteria specific to each data source. It provides interactive network visualization tool for the user to identify an integrated network with a desirable balance between network size and quality. With EnRICH, biologists have an automated yet flexible integration tool to carry out their data analysis and effectively prioritize candidate genes for further investigation.

## Availability and requirements

**Project name:** EnRICH (see Additional file 4: for the jar file of EnRICH program).

**Project home page:**http://xiazhang.public.iastate.edu/ or the software category on the lab homepage http://serb.public.iastate.edu.

**Operating system(s):** platform-independent.

**Programming language:** Java.

**Other requirements:** Java 1.4.2 or higher.

**License:** GNU General Public License.

**Any restrictions to use by non-academics:** NO.

## Competing interest

The author(s) declare that they have no competing interests.

## Authors’ contributions

XZ, JMS and MHWG conceived and designed this software, and drafted this manuscript. XZ coded this software and conducted case study presented in this manuscript. All authors read and approved the final manuscript.

## Supplementary Material

Additional file 1 **Gene lists and their filtration criteria prior to integration. **This file includes a supplementary table that displays names and descriptions of gene lists for integration in case study and some further explanation on the sources of gene lists. Click here for file

Additional file 2 **Gene candidates resulting from the EnRICH filtration and prioritization analyses. **This file is the supplementary table of the five gene candidates from the prioritization by using EnRICH in the case study. Click here for file

Additional file 3 **Description of data processing. **This file includes detail description of data pre-processing for case study, and analysis of the significance of case study result. Click here for file

Additional file 4 **EnRICH program. **This file is the archive file of EnRICH which aggregates Java class files and associated metadata and resources. Click here for file
