# Supplementary material for: EnRICH: Extraction and Ranking using Integration and Criteria Heuristics
Source: BMC Syst Biol. 2013 Jan 15;7:4. doi: 10.1186/1752-0509-7-4 (PMC3564850; doi:10.1186/1752-0509-7-4)
Supplement: Additional file 1 — Gene lists and their filtration criteria prior to integration. This file includes a supplementary table that displays names and descriptions of gene lists for integration in case study and some further explanation on the sources of gene lists. [file 1752-0509-7-4-S1.pdf]

Supplementary Table 1. Gene Lists for integration and criteria on them for filtration

Gene List	Gene List Description	Attribute	Filter on attribute	Potential candidate genes are expected to be
Wt_Nrl.txt	Genes coexpressed with Nrl across all five time points (E16, P2, P6, P10, 4-WEEK) in wild type	Spearman correlation coefficient	>0.9	Present
Wt_Nr2e3.txt	Genes coexpressed with Nr2e3 across all five time points (E16, P2, P6, P10, 4-WEEK) in wild type	Spearman correlation coefficient	>0.9	Present
Wt_Rho.txt	Genes coexpressed with Rho across all five time points (E16, P2, P6, P10, 4-WEEK) in Nrl-mutant.	Spearman correlation coefficient	>0.9	Present
Nrl_Nrl.txt	Genes coexpressed with Nrl across all five time points (E16, P2, P6, P10, 4-WEEK) in Nrl-mutant.	Spearman correlation coefficient	>0.9	Absent
Nrl_Nr2e3.txt	Genes coexpressed with Nr2e3 across all five time points (E16, P2, P6, P10, 4-WEEK) in Nrl-mutant.	Spearman correlation coefficient	>0.9	Absent
Nrl_Rho.txt	Genes coexpressed with Rho across all five time points (E16, P2, P6, P10, 4-WEEK) in Nrl-mutant	Spearman correlation coefficient	>0.9	Absent
Deg_Up.txt	Up-regulated genes (Nrl-mutant VS. Wild type)	Time points at which genes are differentially expressed	P6, P10	Present
Deg_Down.txt	Down-regulated genes (Nrl- mutant VS. Wild type)	Time points at which genes are differentially expressed	P6, P10	Present

Note: The GEO dataset GSE4051 [1] is used to prepare differentially expressed gene lists and coexpressed gene lists. Differentially expressed genes between wild type and Nrl-mutant at four developmental time points (E16, P2, P6 and P10) are obtained with the cut-off q-value controlled at 0.05 (For details, see Additional file 3). Coexpressed genes of Nrl/Nr2e3/Rho are obtained by calculating Spearman correlation coefficients between Nrl/Nr2e3/Rho and other

genes across the five time points (developmental stages) from wild type and Nrl-mutant. R [2] and R packages (affy [3], limma [4], qvalue [5]and doBy[6]) are used to do the computation.

All lists in the above table can be downloaded from http://xiazhang.public.iastate.edu/demo.html

Reference

1. Akimoto M, Cheng H, Zhu D, Brzezinski JA, Khanna R, Filippova E, Oh EC, Jing Y, Linares JL, Brooks M *et al*: **Targeting of GFP to newborn rods by Nrl promoter and temporal expression profiling of flow-sorted photoreceptors**. *Proc Natl Acad Sci U S A* 2006, **103**(10):3890-3895.
2. **R** [http://www.r-project.org/]
3. **affy** [<http://www.bioconductor.org/packages/release/bioc/html/affy.html>]
4. **limma** [<http://www.bioconductor.org/packages/release/bioc/html/limma.html>]
5. **qvalue** [<http://www.bioconductor.org/packages/release/bioc/html/qvalue.html>]
6. **doBy** [http://cran.r-project.org/web/packages/doBy/index.html]
