# Supplementary material for: EnRICH: Extraction and Ranking using Integration and Criteria Heuristics
Source: BMC Syst Biol. 2013 Jan 15;7:4. doi: 10.1186/1752-0509-7-4 (PMC3564850; doi:10.1186/1752-0509-7-4)
Supplement: Additional file 3 — Description of data processing. This file includes detail description of data pre-processing for case study, and analysis of the significance of case study result. [file 1752-0509-7-4-S3.pdf]

The Differentially Expressed Genes (DEGs) of GSE 4051

GSE 4051 [1] is a dataset of Affymetrix arrays. We downloaded the dataset GSE4051_RAW.tar from NCBI database Gene Expression Omnibus (GEO) [2]. We used R Packages to analyze this dataset. The package 'affy' [3] is used to read and pre-process the raw data. The package 'limma' [4] is used to fit the linear model (see below), and compute empirical Bayes moderate t-statistics. In order to control multiple testing FDR (False Discovery Rate) at 5%, we also used the package 'qvalue' [5] to compute q-values and set the cut-off as 0.05.

Linear model

GSE 4051 [1] profiles gene expression in isolated rod photoreceptors at five developmental stages (E16, P2, P6, P10 and 4-weeks) from wild type and *Nrl*-knockouts. We are interested in what genes respond differently between wild type and *Nrl*-knockouts at each of the four developmental stages (E16, P2, P6 and P10). So our linear model takes the mathematical form:

$$Y_{ijk} = \mu + g_i + t_j + gt_{ij} + s_k + e_{ijk}$$

Where the responsible variable Y_{ijk} represents signal intensity for genotype *i*, time point *j* and biological replication *k*. μ denotes the average effect, g_i is the fixed effect of genotype, t_j is the fixed effect of developmental stage, gt_{ij} indicates the interaction between genotype *i* and developmental stage *j*, and e_{ijk} is the random error.

The Significance of the Case Study Results

We found 272 unique differentially expressed genes from the analysis of GSE 4051 [1]. The exclusion of three genes (*Nrl*, *Nr2e3* and *Rho* which were bait genes) results in a candidate pool of 269 genes. By using EnRICH to prioritize this candidate pool, we obtained five genes with the highest priority, and four of them are confirmed retinal disease genes. According to our search for Retinal Disease Genes (see below), only one previously known retinal disease gene (*Rbp3*) was not re-discovered by EnRICH. The Fisher's exact test (see below) also indicates retinal disease genes are significantly enriched in the fived prioritized candidate genes.

Search for Retinal Disease Genes

We used NCBI database Genetics Home Reference [6] to search for the list of documented genes that contribute to the genetic conditions of Retinitis Pigmentosa (Retinitis pigmentosa is a retinal disease caused by abnormalities of the photoreceptor rods and cones or the retinal pigment epithelium). Genes prioritized by EnRICH were compared to this list to determine if they were known retinal disease genes.

Fisher's exact test

We performed a Fisher's exact test to check for enrichment of retinal disease genes in the high priority genes identified using EnRICH.

		Known as retinal disease genes?		
		Yes	No	
Identified as high priority by EnRICH?	Yes	4_d	1_e	5_b
	No	1_f	263_g	264_c
		5_h	264_k	269_a

a: the total number of genes in the candidate pool
b: the total number of genes selected by EnRICH
c: the total number of genes not selected by EnRICH
d: the number of genes that are selected by EnRICH as well as known as retinal disease genes
e: the number of genes that are selected by EnRICH yet not known as retinal disease genes
f: the number of genes that are not selected by EnRICH yet known as retinal disease genes
g: the number of genes that are neither selected by EnRICH nor known as retinal disease genes
h: the total number of genes that are known as retinal genes in the candidate pool
k: the total number of genes that are not known as retinal genes in the candidate pool

Test result computed by R:

p-value = 1.168e-07
Alternative hypothesis: true odds ratio is not equal to 1
95 percent confidence interval:
3.806447e+01 4.503600e+15

The p-value for this test is extremely small, from which we can reject the null hypothesis that retinal disease genes are equally represented between genes selected by EnRICH and genes not selected by EnRICH. Alternatively, the test result favors the hypothesis that retinal disease genes are overrepresented in genes selected by EnRICH.

References

1. Akimoto M, Cheng H, Zhu D, Brzezinski JA, Khanna R, Filippova E, Oh EC, Jing Y, Linares JL, Brooks M *et al*: **Targeting of GFP to newborn rods by Nrl promoter and temporal expression profiling of flow-sorted photoreceptors**. *Proc Natl Acad Sci U S A* 2006, **103**(10):3890-3895.
2. **Gene Expression Omnibus** [<http://www.ncbi.nlm.nih.gov/geo/>]
3. **affy** [<http://www.bioconductor.org/packages/release/bioc/html/affy.html>]
4. **limma** [<http://www.bioconductor.org/packages/release/bioc/html/limma.html>]
5. **qvalue** [<http://www.bioconductor.org/packages/release/bioc/html/qvalue.html>]
6. **Genetics Home Reference** [<http://ghr.nlm.nih.gov/>]
